# Whole-Genome Sequence of *Stenotrophomonas maltophilia* ZBG7B Reveals Its Biotechnological Potential

**DOI:** 10.1128/genomeA.01442-15

**Published:** 2015-12-10

**Authors:** Kok-Gan Chan, Teik-Min Chong, Tan-Guan-Sheng Adrian, Heng Leong Kher, Kar-Wai Hong, Catherine Grandclément, Denis Faure, Wai-Fong Yin, Yves Dessaux

**Affiliations:** a Division of Genetics and Molecular Biology, Institute of Biological Sciences, Faculty of Science, University of Malaya, Kuala Lumpur, Malaysia; b Département de microbiologie, Institut de biologie intégrative de la cellule (Institute of Integrative Cell Biology), CNRS, Gif-sur-Yvette CEDEX, France

## Abstract

*Stenotrophomonas maltophilia* ZBG7B was isolated from vineyard soil of Zellenberg, France. Here, we present the draft genome sequence of this bacterial strain, which has facilitated the prediction of function for several genes encoding biotechnologically important enzymes, such as xylosidase, xylanase, laccase, and chitinase.

## GENOME ANNOUNCEMENT

*Stenotrophomonas maltophilia* has been regarded as one of the promising candidates for biotechnological applications as it has been reported to enhance plant growth (1), possess bioremediation and biotransformation functions (2, 3), and serve as a potential biocontrol agent (4). In this study, *S. maltophilia* strain ZBG7B was isolated from vineyard soil of Zellenberg, France, followed by whole-genome sequencing in order to investigate its genetic architecture.

Genomic DNA was extracted using Epicenter MasterPure DNA purification kit (Epicenter, Inc., USA) according to the manufacturer’s recommendations (5). The sequencing library was constructed using the Illumina Nextera DNA sample prep kit (Illumina, USA), followed by quantification using Qubit version 2.0 (Invitrogen, USA) (6). The library was sequenced using Illumina HiSeq 2500 (Illumina, USA) (7). The raw reads generated upon sequencing were evaluated using FastQC version 0.11.3 (8), followed by trimming and assembly using CLC Genomics Workbench version 7.5 (9). The genomic sequence was then annotated using NCBI Prokaryotic Annotation Pipeline version 2.9 (10).

The draft genome of *S. maltophilia* strain ZBG7B was assembled into 145 contigs with an *N*_50_ of 50,104 bp. The average sequence coverage was 39.2×. The resulting draft genome was approximately 4.065 Mb with a G+C content of 66.3%. The annotation pipeline showed that this strain possesses a total of 3,653 coding sequences, including 3,515 protein-coding genes, 4 rRNA-coding genes, 56 tRNA-coding genes, and 1 gene-encoding other RNA. There are a total of 77 pseudogenes.

The *S. maltophilia* strain ZBG7B genome contains a repertoire of biodegradation-related genes, such as xylosidase (WP_042612111.1), which catalyzes the hydrolysis of xylose residues from the nonreducing ends of xylooligosaccharides (11); xylanase (WP_042612114.1), which depolymerizes xylan into monomeric pentosan (12); and xylose isomerase (WP_042612414.1), which catalyzes the interconversion of D-xylose and D-xylulose, as well as glucose to fructose (13). Furthermore, it also possesses laccase (WP_042613439.1), which is capable of decolorizing synthetic dyes (14), as well as chitinase (WP_042615338.1), which makes this strain a potential biocontrol agent against fungus (15).

### Nucleotide sequence accession numbers.

This whole-genome shotgun project has been deposited at DDBJ/EMBL/GenBank under the accession number JXIP00000000. The version described in this paper is the first version, JXIP01000000.
